# Occurrence, Bioaccumulation, and Human Exposure Risk of the Antiandrogenic Fluorescent Dye 7-(Dimethylamino)-4-methylcoumarin and 7-(Diethylamino)-4-methylcoumarin in the Dongjiang River Basin, South China

**DOI:** 10.3390/toxics12120925

**Published:** 2024-12-20

**Authors:** Yufeng Lai, Yin Huang, Danlin Yang, Jingchuan Xue, Runlin Chen, Rundong Peng, Siying Zhang, Yufei Li, Guochun Yang, Yuxian Liu

**Affiliations:** 1 Key Laboratory of Ministry of Education for Water Quality Security and Protection in Pearl River Delta, School of Environmental Science and Engineering, Guangzhou University, Guangzhou 510006, China; laiyufeng0930@163.com (Y.L.); 18302094293@163.com (Y.H.); 15113127866@163.com (R.C.); 15514040260@163.com (R.P.); 13535217436@163.com (S.Z.); 13538850701@163.com (Y.L.); 2 Guangdong Basic Research Center of Excellence for Ecological Security and Green Development, Key Laboratory for City Cluster Environmental Safety and Green Development of the Ministry of Education, School of Ecology, Environment and Resources, Guangdong University of Technology, Guangzhou 510006, China; 13413933628@163.com (D.Y.); xue@gdut.edu.cn (J.X.); 3 Department of Psychological and Brain Sciences, University of Iowa, Iowa City, IA 52242, USA; guochun-yang@uiowa.edu

**Keywords:** coumarins, environmental occurrence, aquatic ecosystem, bioaccumulation, human exposure, antiandrogenic activity

## Abstract

Recently, 7-diethylamino-4-methylcoumarin (DEAMC) has been identified as a potent antiandrogenic compound in the surface water; however, little is known about the antiandrogenic potentials of other synthetic coumarins and their occurrence in the aquatic ecosystem. In this study, for the first time, we observed that 7-dimethylamino-4-methylcoumarin (DAMC) elicited androgen receptor (AR) antagonistic activity with a 50% inhibitory concentration (IC_50_) of 1.46 µM, which is 14.3 times more potent than that observed for DEAMC (IC_50_ = 20.92 µM). We further collected abiotic (water and sediment) and biotic (plant, plankton, and fish) samples (n = 208) from a subtropical freshwater ecosystem, the Dongjiang River basin, in southern China, and determined the concentrations of the two coumarins in these samples. Overall, DAMC was the predominant compound found in the sediment, plant, algae, zooplankton, and fish muscle samples, with median concentrations at 0.189, 0.421, 0.832, 0.798, and 0.335 ng/g dry wt. (DW), respectively, although it was not detected in any surface water sample. For DEAMC, the median concentrations observed in the surface water, sediment, plant, algae, zooplankton, and fish muscle samples were 0.105 ng/L, 0.012, 0.051, 0.009, 0.008, and 0.181 ng/g DW, respectively. The bioaccumulation factor (BAF) values of DAMC and DEAMC in the algae, zooplankton, and fish muscle exceeded 5000 L/kg, suggesting that the two coumarins may have significant bioaccumulation potentials in aquatic biota. Additionally, the mean daily intake (EDI) of coumarins through fish consumption was estimated as 0.19 ng/kg BW/day for male toddlers. This is the first field study to illustrate the antiandrogenic potential of DAMC and document the widespread occurrence of the two synthetic coumarins in aquatic ecosystems.

## 1. Introduction

Coumarin derivatives have been found in a variety of natural sources such as tonka beans, liquorice, and cassia cinnamon [1]. These chemicals have been used in a broad array of consumer products including air fresheners due to their olfactory properties [2]. In addition, derivatives of coumarin have often been synthesized to meet specific purposes. For example, 7-aminocoumarins, substituted at position 7 with an electron-donating group, exhibit strong fluorescence and have found a broad range of applications in various industrial and consumer products including dyes, optical brighteners, and fluorescent probes [3]. Recently, 7-diethylamino-4-methylcoumarin (DEAMC), one of the 7-aminocoumarins, has demonstrated antiandrogenic potential in both in vitro and in vivo studies [4,5], which raises concerns over the safety of coumarins with similar chemical structures.

Androgens play a crucial role in regulating testis development, spermatogenesis, secondary sexual characteristics, and reproductive behaviors in male organisms through their interaction with the androgen receptor (AR) [6]. Chemicals that inhibit AR activity in males can disrupt reproductive behaviors, reduce fertility, and, in severe cases, lead to gonadal intersex conditions [7,8,9]. Among the major groups of antiandrogenic substances, exposure to phthalates has been linked to various disorders in male reproductive development, including hypospadias, cryptorchidism, reduced testis and penis size, abnormalities in the vas deferens and epididymis, decreased anogenital distance, and the presence of multinucleated germ cells (MNGs) [10,11,12,13]. Over the past 50 years, global sperm counts have reportedly declined by approximately 50%, dropping from 113 million/mL to 66 million/mL (equivalent to an approx. 1% reduction per year), accompanied by a significant increase in sperm morphology and motility abnormalities [14]. The molecular mechanisms underlying these effects vary between different chemicals. For instance, di-n-butyl phthalate (DBP) has been shown to increase anti-Müllerian hormone (AMH) levels, which can impair the development and maturation of adult Leydig cells, alter their final population, or reduce their steroidogenic capacity [15]. In contrast, mono-(2-ethylhexyl) phthalate (MEHP) has been observed to induce germ cell apoptosis and decrease AMH mRNA expression [16].

A recent study has illustrated that wastewater treatment is continuously discharging DEAMC and its transformers in the grams per day range into the Holtemme River, Germany, and the exposure concentrations of DEAMC are in the low micrograms per liter range downstream of the river [17]. Coumarins are slightly soluble in water, which makes them easy to be accumulated in the sediment and biological organisms in the aquatic ecosystem. Hydrophobic interactions are considered as the driving force of partitioning for coumarins between different matrices. Based on the experimental evaluation, the log partitioning coefficients of DEAMC between sediment organic carbon and porewater ranged between 3.2 and 4.2 ng/kg TOC, with initial water concentrations of DEAMC ranging from 0.2 to 5 ug/L. Therefore, the attenuation of coumarins in the river is mainly caused by dilution not transformation, indicating the high persistence of coumarins in the wastewater treatment and aquatic environment [17]. The bioaccumulation of DEAMC in gammarids was also observed at a steady state [17]. However, information regarding the occurrence and bioaccumulation of coumarins in the aquatic environment is limited.

In this work, we firstly identified 7-(dimethylamino)-4-methylcoumarin (DAMC), another commonly used coumarin, as an antiandrogen using a yeast detection system. Then, we determined the concentrations of DEAMC and DAMC in both abiotic (water and sediment) and biotic (plants, plankton, and fish) samples collected from the Dongjiang River basin in southern China. The aims of this work were to (1) determine the exposure levels of DEAMC and DAMC in the abiotic and biotic samples in the freshwater ecosystem; (2) compare the bioaccumulation potentials of coumarins in the biotic samples; and (3) evaluate the potential dietary doses of male exposure to coumarins through fish consumption.

## 2. Materials and Methods

### 2.1. Standards and Reagents

Two target analytes, 7-(dimethylamino)-4-methylcoumarin (sublimation-purified) (DAMC) and 7-diethylamino-4-methylcoumarin (sublimation-purified) (DEAMC), were analyzed in this study. Detailed information on the physicochemical characteristics of these compounds is provided in Table S1. Internal standard linuron-d_6_ was purchased from TRC (North York, Toronto, ON, Canada). DAMC and DEAMC were purchased from TCI (Shanghai, China). The purities of the chemicals were greater than 97%. All organic solvents employed in the analysis were of HPLC grade, procured from Fisher Scientific (Andover, MA, USA). Ultra-pure Milli-Q water was produced using a Milli-Q system (Elga Veolia, High Wycombe, UK).

### 2.2. Yeast Two-Hybrid Assay

The androgen receptor (AR) antagonistic and agonistic activities of DAMC and DEAMC were evaluated using the yeast two-hybrid assay system with a human AR, as described in a previous study [18]. This assay system was generously provided by Peking University. Dihydrotestosterone (DHT) and flutamide (FLU) served as the positive control agonist and antagonist, respectively, in the AR activity assays. The 50% effective concentration (EC_50_) for DHT and the 50% inhibitory concentration (IC_50_) for FLU were determined to be 3.81 nM and 11.18 µM, respectively.

### 2.3. Sample Collection

Detailed information regarding sample collection has been well documented in earlier studies [19,20]. Briefly, a total of 208 samples, including 51 surface water samples, 32 sediment samples, and 123 biological samples (8 algae, 14 aquatic plants, 12 zooplankton, and 89 fish species), were collected from October to November 2020 from the Dongjiang (DJ) River as well as its tributaries including the Xizhijiang (XZJ) River, Shima (SM) River, and Lijiang (LJ) River. All the biological samples were from the DJ River and transported to the laboratory on dry ice after collection. Prior to analysis, water samples were preserved at 4 °C, while other samples were kept at −20 °C. Detailed information about the fish species is provided in the Supplementary Material.

### 2.4. Sample Preparation

#### 2.4.1. Surface Water

The protocol used for the surface water pretreatment was the same as those reported in earlier studies [21,22,23]. Briefly, one liter of surface water was purified with the Oasis MCX^®^ SPE cartridges (500 mg, 6 mL) after separating the particulate matter using glass fiber filter paper (1.2 μm × 55 mm, Whatman, Metstone, UK). Linuron-d_6_ was used as the internal standard. Detailed information about the analytical protocol is provided in the Supplementary Material.

#### 2.4.2. Sediment

Detailed information about the sediment extraction protocol has been provided elsewhere [24,25]. Briefly, 80 mg of the freeze-dried sediment was weighed and transferred to a 15 mL polypropylene (PP) tube, followed by the addition of 10 ng of Linuron-d_6_. Subsequently, target compounds in the sample were extracted using 5 mL of methanol/water mixture (5:3, *v/v*). Other information is provided in the Supplementary Material.

#### 2.4.3. Aquatic Biota

Detailed information regarding the analytical protocol of aquatic biota samples has been provided elsewhere [26,27]. Firstly, lyophilized samples (algae, plant, zooplankton, and fish muscle samples) were pulverized and homogenized. Next, 50 mg of the sample was transferred into a 15 mL PP tube, followed by the addition of 10 μL of Linuron-d_6_ (1.0 mg/L). Then, the sample was extracted with 5 mL of acetone and 2 mL of methanol/acetonitrile mixture (1:1, *v/v*). Other information is provided in the Supplementary Material.

### 2.5. Instrumental Analysis

Target analytes were determined in positive ion multiple reaction monitoring (MRM) mode using an ultra-high-performance liquid chromatographer interfaced with a tandem QTRAP 5500 (MS/MS, SCIEX Technologies, MA, USA) triple quadrupole mass spectrometer. Detailed information regarding the ion source parameters, mass spectrometry parameters, and gradient flow conditions can be found in the Supplementary Material, Tables S2 and S3.

### 2.6. Quality Control and Quality Assurance

The quantification of coumarins in the samples was carried out with an internal standard method based on the response values of linuron-d_6_. A 10-point calibration curve with a regression coefficient (r) >0.99 was established for the target compounds over the concentration range of 0.01–10 ng/mL. In this work, the limit of quantification (LOQ) was calculated as 10 times the signal-to-noise ratio (S/N). Specifically, LOQ values varied depending on the sample type: for surface water, they ranged between 0.003 and 0.006 ng/L; for sediment, the range was from 0.001 to 0.035 ng/g dry weight (DW); plant samples exhibited values between 0.001 and 0.054 ng/g DW; plankton showed a range of 0.002 to 0.174 ng/g dry weight; and for fish muscle, the LOQ values ranged from 0.008 to 0.041 ng/g DW (Table 1). Detailed protocols and information for quality assurance and quality control are provided in the Supplementary Material, Table S4.

### 2.7. Bioaccumulation Capacity Estimation

The bioaccumulation factor (BAF) was used to evaluate the bioaccumulation potential of analytes in aquatic organisms, which was calculated using the following equation [28,29]. Generally, BCFs ≥ 5000 are considered as “highly bioaccumulative”, ≥2000 are considered as “bioaccumulative”, and ≥1000 are considered to have significant bioaccumulation potential.

(1) $$BAF=\frac{C_{Fish/Zooplankton/Algae}}{C_{Total}}\times 1000$$

where $C_{Fish}$, $C_{Zooplankton}$, $C_{Algae}$ and $C_{Total}$ represent the average concentration of the target analyte in fish, zooplankton, algae, and the bulk water, respectively. Notably, for the calculation of BAFs in fish muscle and other biological samples, dry weight-normalized concentrations were used.

### 2.8. Human Exposure Risk Estimation

Freshwater fish has been an important dietary source for people living along the DJ River. To estimate the level of exposure to coumarin from fish intake by males, the estimated dietary intake (EDI) was calculated using the following equation:

(2) $$EDI=\frac{C_{Fish}\times DC}{BW}$$

where $C_{Fish}$ refers to the concentration of the target compounds in fish muscle (ng/g), DC represents the daily fish consumption rate (g/day), and BW represents the body weight of males (kg). For $C_{Fish}$, average and high exposure scenarios were evaluated based on mean and 95th percentile concentrations, respectively. The values of DC were obtained from the Nutrition Data Yearbook of the Chinese Centre for Disease Control and Prevention [30]. The BW data of various age groups were obtained from the Chinese Population Exposure Parameters Manual [31,32]. In this work, an 80% moisture content value was used to convert dry weight-based concentrations to wet weight-based concentrations [33,34].

Further, the health risk posed to males from exposure to these chemicals via fish consumption was evaluated using the Hazard Quotient (HQ), and the HQ was calculated with the following equation:

(3) $$HQ=\frac{EDI}{ADI}$$

where ADI refers to the acceptable daily intake (ADI); HQ > 1 indicates a potential health risk. Based on the data from the European Food Safety Authority, the ADI for coumarin is less than 0.07 mg/kg BW/day [35,36].

### 2.9. Statistical Analysis

Peak integration and quantification were performed using Sciex OS software version 2.2.0.5738 (Applied Biosystems, Foster City, CA, USA). Statistical analyses were performed using Microsoft Excel 2016 and SPSS version 27.0. To facilitate the calculation of basic descriptive statistics, those values below the LOQ were replaced with LOQ/√2. The normality of the data was assessed using the Kolmogorov–Smirnov test. One-way ANOVA was used to assess the differences between groups of data following normal distribution; otherwise, the Mann–Whitney U test was used. To examine correlations between data groups, the Pearson correlation coefficient was used for normally distributed data, while Spearman rank correlation was utilized for non-normal distributions. A *p*-value of less than 0.05 was considered statistically significant.

## 3. Results and Discussions

### 3.1. AR Antagonistic Activities of Coumarins

**Figure 1:**
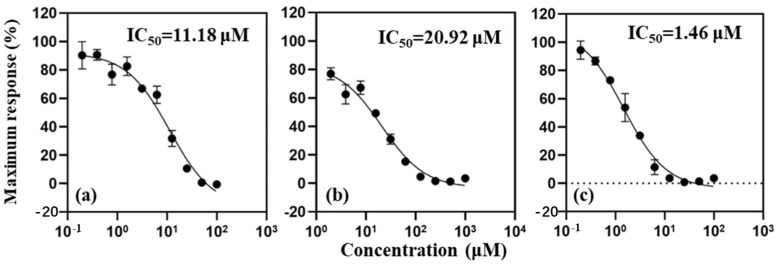
AR antagonistic activities of flutamide (FLU) (**a**), 7-diethylamino-4-methylcoumarin (DEAMC) (**b**), and 7-(dimethylamino)-4-methylcoumarin (DAMC) (**c**). FLU is used as the positive antagonist chemical for the AR antagonist activity assay.

As previously reported, DEAMC did not exhibit any androgen receptor (AR) agonistic activity, and its 50% inhibitory concentration (IC_50_) was determined to be 20.92 μM in this study (Figure 1) [17]. Notably, DAMC demonstrated significantly stronger AR antagonistic activity compared to DEAMC, with an IC_50_ value approximately 7.7 times more potent than that of the reference compound flutamide (11.18 μM) (Figure 1). No AR agonistic activity for DAMC was observed in this study.

### 3.2. Coumarins in Abiotic Samples

#### 3.2.1. Surface Water

DAMC was not detected in any water samples; however, DEAMC was detected in all samples except those from the LJ River, in which the detection rate (DR) was 31.4%. The median concentration of DEAMC was 0.105 ng/L, with the highest concentration found at 0.309 ng/L in the XZJ River (Table 1), which is around three orders of magnitude lower than that measured in the effluent of the Silstedt Wastewater Treatment Plant (0.5 μg/L) [2]. However, in an earlier study performed in 2003, DEAMC was not observed in surface waters in northern Italy [37].

#### 3.2.2. Sediment

DAMC was detected in 12 samples with a DR of 37.5%; DEAMC was detected in 19 samples with a DR of 59.4% (Table 1). The highest and median concentrations of DAMC were 0.668 and 0.189 ng/g DW, respectively; the highest and median concentrations of DEAMC were 0.173 and 0.012 ng/g DW, respectively.

### 3.3. Coumarins in Aquatic Biota

#### 3.3.1. Plants

Both DAMC and DEAMC were detected in 14.3% of plant samples (Table 1). The median concentrations of DAMC and DEAMC in plant samples were 0.421 and 0.051 ng/g DW, respectively. The highest concentration of DAMC was found at 0.781 ng/g DW, and the highest concentration of DEAMC was observed at 0.096 ng/g DW.

#### 3.3.2. Algae

DAMC and DEAMC were found in the alga samples, with DRs being 75% and 62.5%, respectively (Table 1). The median concentrations of DAMC and DEAMC in alga samples were 0.832 and 0.009 ng/g DW, respectively. The highest concentration of DAMC was found at 1.517 ng/g DW, and the highest concentration of DEAMC was observed at 0.031 ng/g DW.

#### 3.3.3. Zooplankton

Both DAMC and DEAMC were detected in 58.3% of zooplankton samples (Table 1). The median concentrations of DAMC and DEAMC were 0.798 and 0.008 ng/g DW, respectively. The highest concentration of DAMC was found at 1.712 ng/g DW, and the highest concentration of DEAMC was observed at 0.118 ng/g DW.

#### 3.3.4. Fish

DAMC and DEAMC were found in fish muscle, with DRs being 30.3% and 50.6%, respectively (Table 1). The median concentrations of DAMC and DEAMC were 0.335 and 0.181 ng/g DW, respectively. The highest concentration of DAMC was found in *Zacco platypus* at 1.574 ng/g DW, and the highest concentration of DEAMC was observed in *Coptodon zillii* at 0.842 ng/g DW.

**Figure 2:**
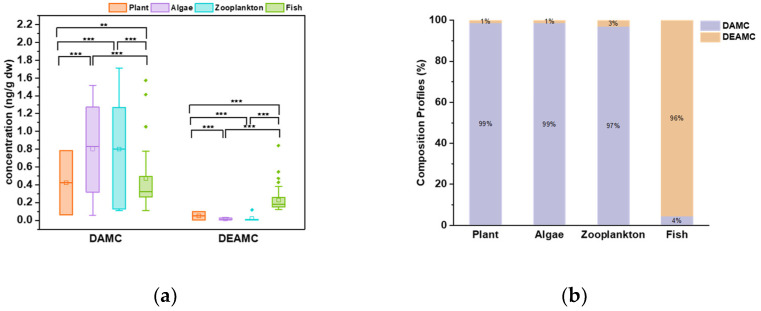
Concentrations of 7-(dimethylamino)-4-methylcoumarin (sublimation-purified) (DAMC) and 7-diethylamino-4-methylcoumarin (sublimation-purified) (DEAMC) in plants, algae, zooplankton, and fish (dry weight basis) (**a**) and compositional profiles of the coumarins in plants, algae, zooplankton, and fish (**b**). Spearman rank correlation coefficients were calculated using logarithmic transformed concentrations; only those samples with both chemicals detected were used in the analysis (*** *p* < 0.001; ** *p* < 0.01).

In summary, significant levels of DAMC and its isomer, DEAMC, were detected across the various aquatic biotic samples in this study. Specifically, as shown in Figure 2a, DAMC was the predominant chemical found in the aquatic biota, contributing to over 97% total concentrations observed in the plant, alga, and zooplankton samples, while in fish muscle, the contributing percent ratio was 65%. We further compared the concentrations of the two chemicals in different types of samples, and the results are shown in Figure 2b. For DAMC, the concentrations in the alga, zooplankton, and fish muscle samples were significantly higher than those observed in the plant samples. And the concentrations of DAMC in the fish muscle were significantly lower than those found in the alga and zooplankton samples. However, for DEAMC, the concentrations in the fish muscle were significantly higher than those detected in the plant, alga, and zooplankton samples.

### 3.4. Bioaccumulation of Coumarins in Aquatic Biota

**Figure 3:**
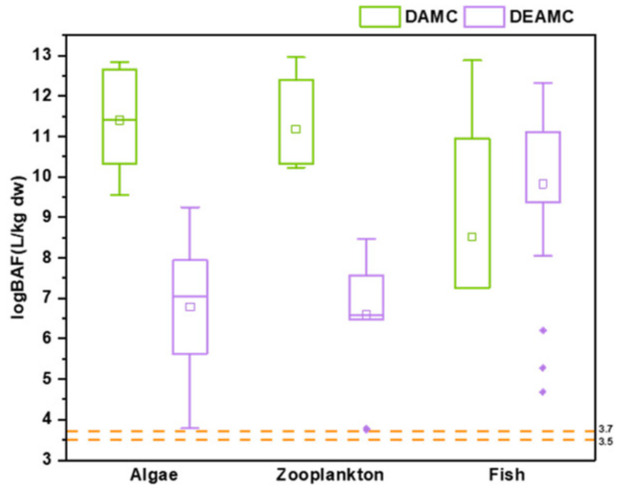
LogBAF values for 7-(dimethylamino)-4-methylcoumarin (sublimation-purified) (DAMC) and 7-diethylamino-4-methylcoumarin (sublimation-purified) (DEAMC) in algae, zooplankton, and fish. The horizontal line in the box plot denotes the median and the small square in the box plot indicates the mean. It should be noted that logBAFs were calculated on the basis of dry weight-normalized concentrations.

To further explore the bioaccumulation capacity of coumarins in aquatic organisms, logBAF (L/kg) was calculated for algae, zooplankton, and fish based on the mean concentrations of these substances. In both algae and zooplankton, the logBAFs of DAMC (11.40 and 11.20 L/kg, respectively) were higher than those calculated for DEAMC (6.78 and 6.59 L/kg, respectively). In fish, the logBAF of DAMC (8.52 L/kg) was lower than that of DEAMC (9.83 L/kg) (Figure 3). Notably, the dry weight-normalized concentrations were used in the calculation, which might lead to an overestimate of the actual values, while fish muscle-based concentrations may underestimate the real values. Thus, the BAF values calculated in this study can be considered as rough estimates. BAF values between 2000 and 5000 (3.3 < logBAF < 3.7) were considered “bioaccumulative”, while BAF values above 5000 (logBAF > 3.7) were considered “very bioaccumulative”. Overall, the logBAF values of both coumarins in all types of aquatic organisms were higher than 3.7, indicating that both DAMC and DEAMC are readily bioaccumulative in aquatic biota.

### 3.5. Estimated Daily Intake of Coumarins Through Fish Ingestion

To enhance the accuracy of estimated daily intake (EDI), the male population was categorized into three age groups: toddlers (2–5 years), children and teenagers (6–17 years), and adults (≥18 years). Considering the high variability in the weight gap among males aged 6–17 years, we further divided children and teenagers (6–17 years) into two age subgroups, children (6–12 years) and teenagers (13–17 years). Daily fish intake amount was obtained from the Chinese Center for Disease Control and Prevention (CDC) *Nutrition Data Yearbook*. It needs to be noted that wet weight-based concentrations were estimated from dry weight-based concentrations when performing EDI calculations. The estimated daily intakes of coumarins for various demographic groups in China are shown in Table 2. The results indicated that toddlers had the highest mean and 95th percentile daily intake of DAMC (0.1 and 0.54 ng/kg BW/day, respectively), followed by children aged 6–12 years (0.07 and 0.41 ng/kg BW/day, respectively), and then by teenagers aged 13–17 years (0.05 and 0.26 ng/kg BW/day, respectively), whose intake levels were similar to those of adults (0.05 and 0.28 ng/kg BW/day, respectively). The daily intake of DEAMC had the same distribution pattern as that of DAMC, with the highest mean and 95th percentile daily intake of 0.09 and 0.28 ng/kg BW/day, respectively, among toddlers, followed by children aged 6–12 years (0.06 and 0.19 ng/kg BW/day, respectively), then teenagers aged 13–17 years (0.04 and 0.14 ng/kg BW/day, respectively), and finally adults (0.05 and 0.15 ng/kg BW/day, respectively). Across all age groups, no significant difference between the exposure doses of DAMC and DEAMC was observed at average exposure levels. However, at high exposure levels, the exposure dose of DAMC was about twice higher than that of DEAMC in all age groups.

To further assess the risk of human exposure to coumarins from fish intake, HQ values were calculated. The results showed that the HQs for both DAMC and DEAMC were less than 1, ranging from 7.03 × 10^−8^ to 7.65 × 10^−7^ and 6.27 × 10^−8^ to 4.00 × 10^−7^, respectively. Therefore, the oral intake of freshwater fish from the Dongjiang River by male residents does not pose a significant risk for coumarin exposure.

## 4. Conclusions

This is the first study reporting the high antiandrogenic activity for DAMC. This paper also determined the concentrations of the two antiandrogenic coumarins, for the first time, in both abiotic (water and sediment) and biotic (plankton, plant, and fish) samples collected from a freshwater ecosystem. Among the matrices analyzed, different distribution patterns were observed for the two coumarins. DAMC was not detected in the surface water; however, it was detected in around 40% of sediment samples, with the highest concentration being 0.668 ng/g DW. A higher concentration of DEAMC was observed in surface water than sediment samples, suggesting that DEAMC is readily distributed in water. Coumarins were also detected in biological samples, with algae and zooplankton samples showing higher concentrations than fish for DAMC, whereas for DEAMC, the highest concentration was observed in the fish muscle samples. We calculated bioaccumulation factors (BAFs) for the target compounds, and the results indicate potential bioaccumulation properties for aquatic organisms. Externally, we assessed the potential human exposure risk of coumarins through the consumption of freshwater fish, with both coumarins posing negligible risk.

## Figures and Tables

**Table 1:** Concentrations of 7-(dimethylamino)-4-methylcoumarin (sublimation-purified) (DAMC) and 7-diethylamino-4-methylcoumarin (sublimation-purified) (DEAMC) in the surface water, sediment, aquatic plants, algae, zooplankton, and fish of the Dongjiang River basin, southern China.

	DR ^a^	Median	Mean	STD ^b^	Max
surface water					
DAMC	-nd ^c^	-nd	-nd	-nd	-nd
DEAMC	31.40%	0.105	0.12	0.097	0.309
sediment					
DAMC	37.5%	0.189	0.247	0.202	0.668
DEAMC	59.4%	0.012	0.038	0.053	0.173
plants					
DAMC	14.29%	0.421	0.421	0.509	0.781
DEAMC	14.29%	0.051	0.051	0.064	0.096
algae					
DAMC	75.00%	0.832	0.804	0.609	1.517
DEAMC	62.50%	0.009	0.015	0.012	0.031
zooplankton					
DAMC	58.33%	0.798	0.802	0.620	1.712
DEAMC	58.33%	0.008	0.023	0.042	0.118
fish muscle					
DAMC	30.30%	0.335	0.467	0.369	1.574
DEAMC	50.60%	0.181	0.230	0.135	0.842

^a^ DR refers to detection rate (%); ^b^ STD refers to standard deviation; ^c^ nd “-” indicated that none of samples were detected.

**Table 2:** Estimated daily intakes (EDI, ng/kg BW/day) of 7-(dimethylamino)-4-methylcoumarin (sublimation-purified) (DAMC) and 7-diethylamino-4-methylcoumarin (sublimation-purified) (DEAMC) in different groups of population in southern China ^a^.

	DAMC	DEAMC
Mean Toddlers (2–5 years)		
	0.10	0.09
Children (6–12 years)	0.07	0.06
Teenagers (13–17 years)	0.05	0.04
Adults (≥18 years)	0.05	0.05
95th percentile		
Toddlers (2–5 years)	0.54	0.28
Children (6–12 years)	0.41	0.19
Teenagers (13–17 years)	0.26	0.14
Adults (≥18 years)	0.28	0.15

^a^ The concentration of coumarins in fish were calculated on a wet weight basis.

## Data Availability

The original contributions presented in the study are included in the article; further inquiries can be directed to the corresponding author.

## Supplementary Materials

The following supporting information can be downloaded at: https://www.mdpi.com/article/10.3390/toxics12120925/s1. Table S1: The physicochemical properties of the target analytes measured in this study (predicted using the US Environmental Protection Agency’s EPI Suite™); Table S2: Retention time, MRM transition, and mass spectrometric parameters of coumarins; Table S3: Major parameters for the gradient flow used in the separation of coumarins; Table S4: Quality assurance/quality control (QA/QC) information of coumarins in each matrix.
