# Supplementary material for: Occurrence, Bioaccumulation, and Human Exposure Risk of the Antiandrogenic Fluorescent Dye 7-(Dimethylamino)-4-methylcoumarin and 7-(Diethylamino)-4-methylcoumarin in the Dongjiang River Basin, South China
Source: Toxics. 2024 Dec 20;12(12):925. doi: 10.3390/toxics12120925 (PMC11728503; doi:10.3390/toxics12120925)
Supplement: Supplementary file 1 [file toxics-12-00925-s001.zip › toxics-3283709-supplementary.pdf]

Supporting Information

Occurrence, Bioaccumulation, and Human Exposure Risk of the Antiandrogenic Fluorescent Dye 7-(dimethylamino)-4-methylcoumarin and 7-(diethylamino)-4-methylcoumarin in the Dongjiang River Basin, South China

Yufeng Lai [1], Yin Huang [1], Danlin Yang [2], Jingchuan Xue [2], Runlin Chen [1], Rundong Peng [1], Siying Zhang [1], Yufei Li [1], Guochun Yang [3] and Yuxian Liu [1,*]

1 Key Laboratory of Ministry of Education for Water Quality Security and Protection in Pearl River Delta, School of Environmental Science and Engineering, Guangzhou University, Guangzhou 510006, China
2 Guangdong Basic Research Center of Excellence for Ecological Security and Green Development, Key Laboratory for City Cluster Environmental Safety and Green Development of the Ministry of Education, School of Ecology, Environment and Resources, Guangdong University of Technology, Guangzhou 510006, China
3 Department of Psychological and Brain Sciences, University of Iowa, Iowa City, IA 52242, USA.
* Correspondence: Email: liuyuxian@gzhu.edu.cn

*Corresponding author

Yuxian Liu, PhD, Guangzhou University, Guangzhou, 510006, China

Email: liuyuxian@gzhu.edu.cn

Submission to: *Toxics*

The Supporting Information contains 6 pages including 4 tables.

Supporting Information Table S1. The physicochemical properties of the target analytes measured in this study (predicted using the US Environmental Protection Agency's EPI Suite™).

Supporting Information Table S2. Retention time, MRM transition, and mass spectrometric parameters of coumarins.

Supporting Information Table S3. Major parameters for the gradient flow used in the separation of coumarins.

Supporting Information Table S4. Quality assurance/quality control (QA/QC) information of coumarins in each matrix.

2. Materials and Methods

2.3. Sample collection

Detailed information regarding sample collection has been well documented in earlier studies [7,8]. Briefly, a total of 208 samples, including 51 surface water samples, 32 sediment samples, and 123 biological samples (8 algae, 14 aquatic plants, 12 zooplankton, and 89 fish species), were collected from October to November 2020 from the Dongjiang (DJ) River as well as its tributaries including the Xizhijiang (XZJ) River, Shima (SM) River, and Lijiang (LJ) River. All the biological samples were from the DJ River and transported to the laboratory on dry ice after collection. Prior to analysis, water samples were preserved at 4°C, while other samples were kept at -20°C.

Among the biological samples, 89 fish muscle specimens were collected, representing 28 different species of freshwater fish, namely, *Ctenogobius giurinus, Mastacembelus aculeatus, Vanmanenia pingchowensis, Hemicculter leucisculus, Channa argus, Culter alburnus, Acrossocheilus parallens, Squalidus argentatus, Oreochromis niloticus, Acrossocheilus beijiangensis, Pseudogobio vaillanti, Cyprinus carpio, Zacco platypus, Rhinogobius giurinus, Gastromyzoninae* sp., *Cirrhinus molitorella, Xenocypris argentea, Hypostomus plecostomus, Coptodon zillii, Opsariichthys bidens, Vanmanenia stenosoma, Rhodeus sinensis, Eleotris oxycephala, Acheilognathus macropterus, Sarcocheilichthys nigripinnis, Carassius auratus, Squaliobarbus curriculus,* and *Puntius semifasciolatus*. A total of 14 stem leaves, representing 7 species of herbaceous aquatic plants, were collected. The species of plants analyzed were *Water hyacinth, Panicum bisulcatum thunb, Wedelia trilobata, Celastrus orbiculatus, Cyperus rotundus* L., *Ludwigia octovalvis,* and *Miscanthus sinensis anderss*.

2.4. Sample Preparation

Surface Water. The protocol used for the surface water pretreatment was the same as those reported in earlier studies [9–11]. Briefly, one liter of surface water was purified with the Oasis MCX® SPE cartridges (500 mg, 6 mL). Firstly, particulate matter was removed from the surface water using glass fiber filter paper (1.2 μm × 55 mm, Whatman, Metstone, UK). Then, 10 ng of Linuron-d_6 was added to the filtered water. Next, the SPE cartridge was cleaned with 6 mL of methanol and conditioned with 6 mL of milli-Q water. Afterwards, the filtered water was passed through the SPE cartridge at a constant flow rate (5 mL/min) and dried under vacuum for 30 min. The extraction was performed using a mixture of 3 mL of methanol and 6 mL of acetonitrile, and the extracts were concentrated under a gentle nitrogen stream. For reconstitution, 500 μL of methanol was used, and prior to instrumental analysis, a 0.22 μm glass fiber filter (Shanghai Xinya Purification Equipment Co., Ltd., Shanghai, China) was applied.

Sediment. Detailed information about the sediment extraction protocol has been provided elsewhere [12,13]. Briefly, 80 mg of the freeze-dried sediment was accurately weighed and transferred to a 15 mL polypropylene (PP) tube, followed by the addition of 10 ng of Linuron-d_6. Subsequently, target compounds in the sample were extracted using 5 mL of methanol/water mixture (5:3, v/v). The sample was shaken for 60 minutes, sonicated for 30 minutes, and then centrifuged at 5000 x *g*. The supernatant was transferred to a clean 15 mL PP tube. This procedure was performed twice, and the extracts were combined and concentrated to 3 mL under a gentle stream of nitrogen. Then, the Oasis MCX®SPE filter cartridge (500 mg, 6 mL) was used to purify 10 mL of ultrapure water containing 0.2% formic acid (v/v). During the purification process, the cartridge was cleaned with 3 mL of methanol and 6 mL of acetonitrile and then conditioned with 10 mL of aqueous solution containing 0.2% formic acid. In total, 10 mL of ultrapure water containing 0.2% formic acid was used to clean the cartridge. After the cleaning was completed, the cartridge was dried under vacuum. Afterwards, the target compounds were eluted from the cartridge by 9 mL of methanol, and the eluate was concentrated to nearly dryness. Then, approximately 500 μL of methanol was used for reconstitution. A 0.22 μm filter paper was used to remove particles prior to instrumental analysis.

Aquatic biota. Detailed information regarding the analytical protocol of aquatic biota samples has been provided elsewhere [14,15]. Firstly, lyophilized samples (alga, plant, zooplankton, and fish muscle samples) were pulverized and homogenized. Next, 50 mg of the sample was transferred into a 15 mL PP tube, followed by the addition of 10 μL of Linuron-d_6 (1.0 mg/L) as an internal standard. Then, the sample was extracted with 5 mL of acetone and 2 mL of methanol/acetonitrile mixture (1:1, v/v). After centrifugation at 5000 x *g* for 15 min, the supernatants were combined and concentrated to near dryness in a mild nitrogen stream. For the reconstitution, a mixture of 500 μL of methanol/acetonitrile/water (3:3:4,v/v/v) was used and the extract was centrifuged at 5000 x *g* for 5 min before instrumental analysis.

2.6 Quality control and quality assurance

The quantification of coumarin in the samples was carried out with an internal standard method based on the response values of linuron-d_6. A 10-point calibration curve with a

regression coefficient (r) > 0.99 was established for the target compounds over the concentration range of 0.01 ~ 10 ng/mL. In this work, the limit of quantification (LOQ) was calculated as 10 times the signal-to-noise ratio (S/N). Specifically, LOQ values varied depending on the sample type: for surface water, they ranged between 0.003 and 0.006 ng/L; for sediment, the range was from 0.001 to 0.035 ng/g dry weight (DW); plant samples exhibited values between 0.001 and 0.054 ng/g DW; plankton showed a range of 0.002 to 0.174 ng/g dry weight; and for fish muscle, the LOQ values ranged from 0.008 to 0.041 ng/g DW (Table 1).

Instrumental and procedural blanks were included in each batch of analyzed samples to monitor potential contamination arising from the glassware and reagents used in the analytical protocol. In this study, the target analytes were not detected in any blank samples. To verify the accuracy, recovery, and precision of the analytical method, evaluations were conducted on the pre-extraction matrix spike, pre-extraction matrix spike duplicates, and post-extraction matrix spike. Each matrix was fortified with 10 ng of the target analytes and subjected to the full analytical procedure. For each sample type, absolute recoveries of the two target compounds were shown as follows: 63.0% in surface water; 50.6% to 68.3% in sediment; 94.1% to 98.0% in fish muscle; and 93.5% to 95.0% in aquatic biota (plants, algae, and zooplankton).

Table S1. The physicochemical properties of the target analytes measured in this study (predicted using the US Environmental Protection Agency's EPI Suite™).

Compound	Abbreviation	CAS #	Structure	M.W.	Log K_{ow} [a]	Solubility (mg/L) at 25 °C[b]	BAF [c]	BCF [d]
7-dimethylamino-4-methylcoumarin (sublimation purified)	DAMC	87-01-4	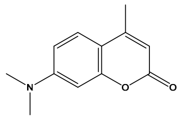	203.24	2.24	516.9	9.661	9.661
7-diethylamino-4-methylcoumarin (sublimation purified)	DEAMC	91-44-1	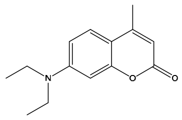	231.29	3.22	53.28	38.09	38.09

[*] Based on experiment database match. [a] Based on KOWWIN™ v1.68 estimate. [b] Based on WSKOWWIN™ v1.42. [c and d] Based on BCFBAF™ v3.01.

Table S2. Retention time, MRM transition, and mass spectrometric parameters of coumarins.

Coumarins	RT[a]	Q1[b]	Q3[c]	DP[d]	CE[e]	EP[f]	CXP[g]
Linuron-d6 1	15.0	255.000	160.000	106.000	27.000	8.000	14.000
Linuron-d6 2	15.0	255.000	185.000	106.000	22.000	8.000	17.000
DEAMC 1	15.0	232.000	188.000	104.000	41.000	5.000	17.000
DEAMC 2	15.0	232.000	203.000	104.000	25.000	5.000	20.000
DAMC 1	15.0	204.000	148.000	150.000	31.000	11.000	14.000
DAMC 2	15.0	204.000	132.000	150.000	48.000	10.000	13.000

[a]RT: Retention time (min). [b]Q1: Precursor ion. [c]Q3: Product ion. [d]DP: Declustering potential. [e]CE: Collision energy. [f]EP: Entrance potential. [g]CXP: Cell exit potential.

Table S3. Major parameters for the gradient flow used in the separation of coumarins.

Time (min)	Flow Rate (mL/min)	Mobile Phase A (%)	Mobile Phase B (%)
0	0.3	98	2
1	0.3	96	4
6	0.3	1	99
8	0.3	1	99
9	0.3	98	2
10	0.3	98	2

*0.1% formic acid in milli-Q water and 0.1% formic acid in acetonitrile were used as mobile phases A and B, respectively.

Table S4. Quality assurance/quality control (QA/QC) information of coumarins in each matrix.[a]

	DAMC	DEAMC
water		
Recoveries (%)	63.00	63.00
Matrix effect (%)	87.90	91.50
Accuracy (%)	55.40	57.70
Precision (%)	12.9	9.6
sediment		
Recoveries (%)	50.60	68.30
Matrix effect (%)	110.00	85.04
Accuracy (%)	61.16	64.30
Precision (%)	1.67	2.62
fish		
Recoveries (%)	97.96	94.14
Matrix effect (%)	74.27	80.05
Accuracy (%)	72.90	75.57
Precision (%)	16.39	14.31
plants, algae, and zooplankton		
Recoveries (%)	93.47	94.93
Matrix effect (%)	120.42	99.07
Accuracy (%)	112.56	94.04
Precision (%)	3.95	3.01

[a] For the pre-extraction matrix spike sample, randomly selected samples ($n > 3$) were spiked with 10 ng of the target analytes and 10 ng Linuron-d6 prior to extraction; for the post-extraction matrix spike sample, the randomly selected samples were spiked with 10 ng Linuron-d6 prior to extraction and 10 ng target analytes prior to reconstitution.
